# Integration of relative metabolomics and transcriptomics time-course data in a metabolic model pinpoints effects of ribosome biogenesis defects on *Arabidopsis thaliana* metabolism

**DOI:** 10.1038/s41598-021-84114-y

**Published:** 2021-02-26

**Authors:** Christopher Pries, Zahra Razaghi-Moghadam, Joachim Kopka, Zoran Nikoloski

**Affiliations:** 1 Metabolomics Infrastructure Group, Max-Planck Institute of Molecular Plant Physiology, 14476 Potsdam, Germany; 2 Bioinformatics, Institute of Biochemistry and Biology, University of Potsdam, 14476 Potsdam, Germany; 3 Systems Biology and Mathematical Modeling Group, Max-Planck Institute of Molecular Plant Physiology, 14476 Potsdam, Germany

**Keywords:** Computational biology and bioinformatics, Plant sciences, Systems biology

## Abstract

Ribosome biogenesis is tightly associated to plant metabolism due to the usage of ribosomes in the synthesis of proteins necessary to drive metabolic pathways. Given the central role of ribosome biogenesis in cell physiology, it is important to characterize the impact of different components involved in this process on plant metabolism. Double mutants of the *Arabidopsis thaliana* cytosolic 60S maturation factors *REIL1* and *REIL2* do not resume growth after shift to moderate 10 $^{\circ }\hbox {C}$ chilling conditions. To gain mechanistic insights into the metabolic effects of this ribosome biogenesis defect on metabolism, we developed TC-iReMet2, a constraint-based modelling approach that integrates relative metabolomics and transcriptomics time-course data to predict differential fluxes on a genome-scale level. We employed TC-iReMet2 with metabolomics and transcriptomics data from the *Arabidopsis* Columbia 0 wild type and the *reil1-1 reil2-1* double mutant before and after cold shift. We identified reactions and pathways that are highly altered in a mutant relative to the wild type. These pathways include the Calvin–Benson cycle, photorespiration, gluconeogenesis, and glycolysis. Our findings also indicated differential NAD(P)/NAD(P)H ratios after cold shift. TC-iReMet2 allows for mechanistic hypothesis generation and interpretation of system biology experiments related to metabolic fluxes on a genome-scale level.

## Introduction

Ribosomes are the workplaces of protein biosynthesis, and defects in the pathway of ribosome biogenesis have an effect on many cellular processes, like metabolism, which critically depend on enzymatic proteins. While metabolism is known to affect ribosome function via the target of rapamycin (TOR) signalling pathway, little is known about how defects in ribosome biogenesis feed back on metabolism^1^. The *Arabidopsis thaliana* REIL proteins are involved in the late cytosolic steps of 60S ribosome subunit maturation and are required for growth under low temperature^2^. The *reil1-1 reil2-1* double mutant is deficient for both REIL paralogs and, unlike *Arabidopsis* Col-0 wild type, does not resume growth after cold shift, even to moderate 10 $^{\circ }$C chilling conditions. This experimental system is ideally suited to investigate the cytosolic ribosome biogenesis defect at the metabolic level, since both wild type and mutant show growth arrest during the early hibernation phase (less than seven days after cold shift) followed by differential growth in the later stages. Therefore, mechanistic insights in the impact of defects of the mutant’s ribosome biogenesis on metabolism may become apparent early after cold shift, during hibernation phase.

One possibility to investigate the feedback of ribosome biogenesis defects on metabolism is the characterization of reaction fluxes. Metabolic fluxes depend, in part, on the metabolite pools^3^. They also depend on the enzymatic setup of a cell, which is in turn governed by gene regulatory and signalling networks that affect protein activity^4^. However, determination of metabolic fluxes is a tedious and labour-intensive task^5–7^. A targeted analysis that predicts relevant fluxes for hypothesis generation based on integration of available high-throughput data sets from systems biology studies may streamline the planning of such time-consuming experimental flux studies.

In this regard, constraint-based approaches have proved as a valuable tool for hypotheses generation regarding flux distributions and their differential behaviour. For instance, the simplest of these approaches, flux balance analysis (FBA), can predict steady-state fluxes in bacteria at exponential growth^8^. In general, metabolic fluxes of a system are predicted under the assumption that this system operates in steady-state and optimizes an objective (e.g. biomass yield). If feasible, the resulting mathematical approach often results in a non-unique flux distribution. To this end, constraints defined through integration of high-throughput data can reduce the solution space of feasible flux distributions^9–11^. Such approaches have been shown to result in a more accurate prediction which is closer to the actual physiological state^12^. Despite the availability of methods that integrate high-throughput data, their full potential has yet to be realized^13^.

Of particular interest are approaches which allow integration of relative metabolite levels, since these datasets are easier to obtain in contrast to absolute metabolite concentrations used in thermodynamic flux balance analysis^14^ as well as approaches that use time-series data (e.g. TREM-Flux^15^, uFBA^16^, and dFBA^17^). iReMet-flux^18^ is the only constraint-based approach to date that can integrate relative metabolite levels to investigate differential flux behaviour between two scenarios. It relies on a mass-action-like description of reaction rates (i.e. flux). In contrast to uFBA, iReMet-Flux does not require data on absolute quantification of metabolite levels and therefore allows for a broader application due to the availability of relative metabolomics data. In contrast to TREM-Flux, it does not assume a linear scaling with the change of metabolite levels between two time points. In addition, iReMet-Flux differs from a recent approach in which the relative metabolomics data are integrated on a qualitative level (i.e. increases or decreases)^14^. Similar to the objective on which MOMA is based^19^, iReMet-flux minimizes the flux differences between two scenarios, but does not rely on pre-calculated flux distributions for a reference scenario. Additionally, iReMet-flux allows for the integration of relative enzyme levels either by direct usage of quantitative or qualitative proteomics data, or via gene expression ratio that can serve as a proxy^10,20,21^. However, if employed to time-series data, it does not account for the magnitude of possible flux changes between time steps. To address this problem, we extended iReMet-flux to account for temporal changes, while keeping the possibility of multi-level high-throughput data integration.

Here, we aimed to develop a novel constraint-based approach, termed TC-iReMet2, that facilitates the integration of relative metabolite and transcript levels while accounting for temporal change of physiological parameters. We used TC-iReMet2 to investigate differential flux behaviour of *A. thaliana* Col-0 wild type and *reil1-1 reil2-1* double mutant plants before and after cold shift. Finally, we provided directly testable hypotheses about the impact of REIL-mediated deficiency in ribosome biogenesis on metabolism.

## Results

### Formulation of TC-iReMet2

We propose Time Course Integration of Relative Metabolite and Transcript levels (TC-iReMet2) that estimates fluxes based on the integration of time-course data on relative metabolite and transcript levels. The key feature of TC-iReMet2 is that it accounts for the possible magnitude of flux changes between time points and thus could provide a more accurate explanation of flux rerouting over time. We show that TC-iReMet2 can be applied to study flux redistributions in pathways in a large-scale metabolic network of *A. thaliana*. Unlike genome-scale metabolic networks^22^, we refer to large-scale models as those reconstructed following a bottom-up approach^23^.

Similar to other constraint-based approaches, TC-iReMet2 uses a stoichiometric matrix *S* of the considered metabolic model. The rows of the stoichiometric matrix correspond to metabolites, and columns stand for reactions. The integer entries denote the molarity of a product (positive entry) or a substrate (negative entry) in a reaction, ensuring mass and charge conservation. In the following, we assume that the investigated metabolic network contains *P* reactions and *n* metabolites, and that its functioning is compared between two experimental scenarios, denoted by *A* and *B* (e.g. mutant and wild type) over to time points ${t+1}$ and *t*. Furthermore, we denote by $p_1$ the number of irreversible reactions and by $p-p_1$ the number of reversible reactions.

Under mass action kinetics, a flux through an irreversible reaction *i*, $1 \le p_1 \le p_1$, can be formally described by $v_i = k_i E_i \prod _{j=1}^n (x_j)^{|S_{ji}|}$, where $x_j$ denotes the concentration of metabolite *j*, $S_{ji}$ denotes the stoichiometric coefficient with which a metabolite *j* enters a reaction *i* as a substrate, $E_i$ denotes the enzyme concentration and $k_i$ denotes the reaction specific rate constant. Note that this expression can be written equally for scenario *A*: $v_i^A = k_i^A E_i^A \prod _{j=1}^n (x_j^A)^{|S_{ji}|}$and scenario *B*: $v_i^B = k_i^B E_i^B \prod _{j=1}^n (x_j^B)^{|S_{ji}|}$, where the rate constant $k_i$ is the only unchanged parameter ($k_i^A=k_i^B$) - as it summarizes the key property of the same enzyme. Therefore, the relationship of a single flux between two scenarios can be written as:

1 $$\begin{aligned} \frac{v_i^A}{v_i^B} = \frac{E_i^A}{E_i^B} \prod _{j=1}^n \frac{(x_j^A)^{|S_{ji}|}}{(x_j^B)^{|S_{ji}|}} \end{aligned}$$

To simplify the notation, we will refer to the ratio of metabolite levels of *j* as $r_j = \frac{x_j^A}{x_j^B}$ and the ratio of enzyme levels catalyzing reaction *i* as $q_i = \frac{E_i^A}{E_i^B}$. This allows us to rewrite the ratio of flux rates of reaction *i* as $\frac{v_i^A}{v_i^B} = q_i \prod _{j=1}^n (r_j)^{|S_{ji}|}$ or equivalently $v_i^A = [q_i \prod _{j=1}^n (r_j)^{|S_{ji}|}] v_i^B$.

Determining the entirety of metabolite and enzyme concentrations is not possible with the existing technologies^24,25^. For metabolite ratios, only a small portion of the metabolome, and hence metabolite ratios, can be quantified. To account for the case that a metabolite ratio cannot be measured, general upper and lower boundaries for metabolite ratios are introduced. If the ratio of metabolite *j* is experimentally quantified, it is indicated by $\chi (r_j) = 1$ and otherwise by $\chi (r_j) = 0$.

In the absence of enzyme ratios, we use the Gene Protein Reaction (GPR) rules of metabolic models to approximate enzyme ratios using transcriptomic data. The GPR roles are defined by a set of Boolean expressions that describe which genes encode an enzyme. For example, gene products encoding for isoenzymes or isoforms are linked by an OR operator. Conversely, protein subunits that must be present simultaneously to form an active enzyme are linked by an AND operator. In case of an enzyme encoded by one gene, the enzyme concentration is approximated by its expression value. For each reaction that is catalyzed by a complex requiring multiple genes, the enzyme concentration is set to the minimum expression value of gene products connected by the AND operator. For the OR operator, the sum of expression values for the respective genes is used. These rules were applied to each reaction in both scenarios, fractioned and assigned as the corresponding enzyme ratio. Therefore, an enzyme ratio is represented by a ratio of gene expression levels following the GPR rules. Equivalently to metabolite ratios, if a GPR rule for reaction *i* is defined, it is indicated by $H (q_i) = 1$ and for reactions without a defined GPR rule, by $H (q_i) = 0$.

In this setup, we only consider constraints for irreversible reactions, since more than 80% of reactions that are assumed to follow mass–action-like kinetics (this excludes artificial and transport reactions) are irreversible in the analyzed model of *A. thaliana*. This has been verified by performing flux variability analysis at a fixed flux through the biomass reaction, to specify that 80% of reactions operate in only one direction^18^. A ratio constraint for reaction *i* is included if not only the enzyme ratio, but also at least one of the substrate ratios corresponding to that reaction is available. For metabolites or enzymes whose ratios could not be determined we use the extremal values found at that specific time point. Let *F*(*i*) denote the set of substrates of reaction *i*. Additionally, let the set of irreversible reactions with at least one experimentally quantified metabolite ratio and approximated enzyme ratio be denoted by $\mathfrak {I}= \{ i | \sum _{j\in F(i)} \chi (r_j)> 0 \;\; \& \; H(q_i) > 0 \}$. A measured metabolite ratio for *j* and transcript ratio of *i* are indicated by ${{\hat{r}}_j^{min}} \le {{\hat{r}}_j} \le {{\hat{r}}_j^{max}}$ and ${{\hat{q}}_i^{min}} \le {{\hat{q}}_i} \le {{\hat{q}}_i^{max}}$, respectively. The bounds are defined as multiples of the standard deviation for the ratio. Cofactors were treated as unmeasured metabolites and for them the lower and upper bounds are $min_{m:\; m \in \{ \ell | \chi (r_\ell )=1\}}\;{{\hat{r}}_m^{min}}$ and $max_{m:\; m \in \{ \ell | \chi (r_\ell )=1\}}\;{{\hat{r}}_m^{max}}$, respectively. Equivalently we can write $min_{m:\; m \in \{ \eta | H (q_\eta )=1\}}\;{{\hat{q}}_m^{min}}$ and $max_{m:\; m \in \{ \eta | H (q_\eta )=1\}}\;{{\hat{q}}_m^{max}}$ for unmeasured transcript ratios. Furthermore, to account for enzymes that are substrate saturated and in turn would lead to infeasibilities due to metabolite ratio constraints, slack variables $\varepsilon _i$ were introduced to relax the strict ratio constraints. To minimize these relaxations a weighting of the summed slack variables of $\epsilon = 0.01$ was used. Hence, a ratio constraint was formulated as follows:

2 $$\begin{aligned} v_i^{B}q_i\prod _{j\in F(i)}(r_j^{min})^{|S_{ji}|}-\varepsilon _i \le v_i^{A} \le v_i^{B}q_i\prod _{j\in F(i)}(r_j^{max})^{|S_{ji}|}+\varepsilon _i. \end{aligned}$$

Similarly, a ratio constraint for the biomass reaction can be formulated. To this end, a time-point specific biomass fraction, denoted by $\varkappa _{t+1}$, can be calculated. First, the maximum biomass yield, denoted by *opt*, is calculated for both scenarios via FBA. A biomass fraction $\varkappa _{t+1}$ between both scenarios is then determined by using proxies for biomass (for a detailed description see Methods - Parameterizing the objection and of TC-iReMet2 and estimating fractions of biomass yield). We fix the biomass reaction of scenario *B* to its respective value derived from FBA. In contrast, biomass flux in scenario *A* is fixed to a fraction $\varkappa _{t+1}$ of its optimal yield. Lower and upper bounds are specified as deviations, denoted by $\delta$, of the calculated fraction. Therefore, biomass fluxes for both scenarios can be constrained as follows:

3 $$\begin{aligned}&(\varkappa _{t+1}-\delta )\;opt^A \le v_{Biomass}^{A} \le (\varkappa _{t+1}+\delta )\;opt^A, \end{aligned}$$

4 $$\begin{aligned}&v_{biomass}^B = opt^B. \end{aligned}$$

Furthermore, we assume that: (i) the metabolic network to operate in quasi-steady state at every time point. Hence, $Sv^A = Sv^B = 0$, where $v^A$ and $v^B$ denote the flux distributions of scenarios *A* and *B* respectively. (ii) the biological system aims to maintain an optimal state given by the enzymatic setup. This assumption is captured by making sure that the flux distributions between the two scenarios at a given time point $t+1$ are as close as possible, i.e. $||(v^{B}_{t+1}-v^{A}_{t+1})||_2^{2}$. (iii) the physiological state at time $t+1$ depends on the physiological state at time *t*. We model this assumption by accounting for the magnitude of possible physiological changes by assuring that the difference of flux distributions between time points is as small as possible, i.e. $||(v^{B}_{t+1}-v^{B}_{t})||_2^{2}, \; ||(v^{A}_{t+1}-v^{A}_{t})||_2^{2}$, respectively. This magnitude obviously depends on the difference between time points, where the magnitude of possible flux changes increases with time. To this end, we introduce weighting factors to minimize the difference of flux distributions between scenarios at the current time point, weighted by $\alpha$, as well as for differences between prior time points for scenario *A*, weighted by $\beta$, and scenario *B*, weighted by $\gamma$.

In summary, the TC-iReMet2 approach is cast as a quadratic program (QP) as follows:

$$\begin{aligned} \begin{array}{ll@{}ll} {\displaystyle \min _{v^{A},v^{B},\varepsilon } \alpha ||(v^{A}_{t+1}-v^{B}_{t+1})||_2^{2}+\beta ||(v^{A}_{t+1}-v^{A}_{t})||_2^{2}+\gamma ||(v^{B}_{t+1}-v^{B}_{t})||_2^{2} + \epsilon \; \Sigma _{i=1}^{p} \;\varepsilon _i} &{} &{} \\ \\ \text {s.t.}&{} &{} &{} \\ &{} &{} &{} \\ \\ {Sv_{t+1}^{A}=Sv_{t+1}^{B} = 0 ,} \\ \\ {v_{min}^{A} \le v^{A}_{t+1} \le v_{max}^{A} ,} \\ \\ {v_{min}^{B} \le v^{B}_{t+1} \le v_{max}^{B} ,} \\ \\ {(\varkappa _{t+1}-\delta )\;opt^A \le v_{Biomass}^{A} \le (\varkappa _{t+1}+\delta )\;opt^A ,} \\ \\ {v_{biomass}^B = opt^B ,}\\ \\ {\forall i \in \mathfrak {I}: v_i^{B}q_{i}\prod _{j\in F(i)}(r_j^{min})^{|S_{ji}|}-\varepsilon _i \le v_i^{A} \le v_i^{B}q_{i}\prod _{j\in F(i)}(r_j^{max})^{|S_{ji}|}+\varepsilon _i ,} \\ \\ {\forall j \in \{ \ell | \chi (r_\ell )=1\}:r^{min}_j=\hat{r}_j^{min}, r^{max}_j=\hat{r}_j^{max} ,} \\ \\ { \forall j \in \{ \ell | \chi (r_\ell )=0\}:r^{min}_\ell =min_{m:\; m \in \{ \ell | \chi (r_\ell )=1\}}\;{{\hat{r}}_m^{min}}, \;\; r^{max}_\ell =max_{m:\; m \in \{ \ell | \chi (r_\ell )=0\}}\;{{\hat{r}}_m^{max}} ,} \\ \\ {\forall i \in \{ \eta | H (q_\eta )=1\}:q^{min}_i=\hat{q}_i^{min}, q^{max}_i=\hat{q}_i^{max} ,} \\ \\ {\forall i \in \{ \eta | H (q_\eta )=0\}:q^{min}_\eta =min_{m:\; m \in \{ \eta | H (q_\eta )=1\}}\;{{\hat{q}}_m^{min}}, \;\; q^{max}_\eta =max_{m:\; m \in \{ \eta | H (q_\eta )=0\}}\;{{\hat{q}}_m^{max}} ,} \\ \\ {\forall i \in \mathfrak {I}: 0 \le \varepsilon _i}. \\ \\ \end{array} \end{aligned}$$

### Application of TC-iReMet2 to data from the *reil1-1 reil2-1 A. thaliana* mutant

**Figure 1:**
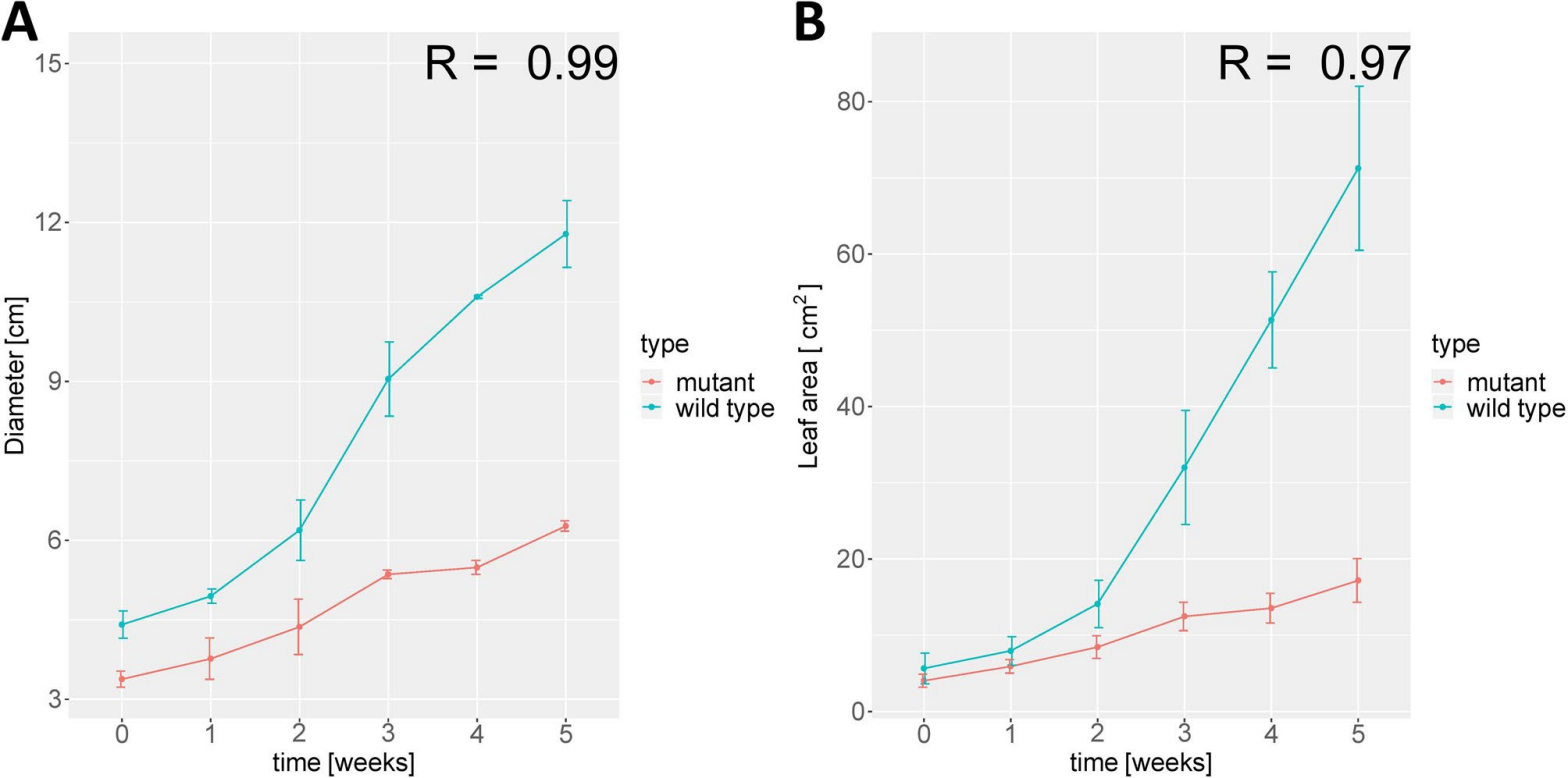
Morphometric analyses of *reil1-1 reil2-1* and wild type after shift from optimized (20 $^{\circ }$C) to low temperatures (10 $^{\circ }$C). *Reil1-1 reil2-1* double mutants and *A. thaliana* Col-0 wild type plants were shifted at developmental stage 1.10^29^. Week-0 plants were grown at 20 $^{\circ }$C and assayed before the temperature shift. Rosette diameter, (**A**); leaf area, (**B**), (mean +/− standard deviation; $n =$3–10 plants), for original data and definitions of morphometric parameters refer to Schmidt et al. 2013^2^. The R coefficients represent the Pearson correlation between mutant and wild type with respect to the Diameter (**A**) (*P*-value = $2.91^{-11}$) and Leaf area (**B**) (*P*-value = $1.53^{-5}$).

We employed TC-iReMet2 to gain insights into the metabolic effects of the ribosome biogenesis defect that is caused by *A. thaliana* REIL deficiency. To this end, we compared predicted flux differences between Col-0 wild type and *reil1-1 reil2-1* double mutant with deficiency in cytosolic 60S ribosome biogenesis. The REIL proteins are required for growth when plants are shifted to cold ($< 10$ $^{\circ }\hbox {C}$) conditions, but not at optimal temperature (${\simeq }20$ $^{\circ }\hbox {C}$)^2^. The *reil1-1 reil2-1* double mutant and wild type differ only slightly in size when grown at 20 $^{\circ }\hbox {C}$. Young developing leaves of the mutants showed an acute tip and two basal serrations instead of the typical rounded leaves of the Col-0 wild type, and were similar to the pointed leaves phenotype of cytosolic ribosome mutants^26–28^. However, the pointed-leaf phenotype of the *reil1-1 reil2-1* double mutant was no longer apparent after transfer to soil and at developmental stages < 1.10^29^ that were analyzed in this study. When shifted to 10 $^{\circ }$C (cold), both the mutant and the wild type stopped growing. Following seven days in the cold, the wild type resumed growth, while the mutant remained strongly growth-inhibited (Fig. 1, Supplementary Table S1). The mutant survived at least four weeks after cold shift and maintained cellular integrity as was determined by electrolyte leakage assays of rosette leaves^30^. Growth parameters of wild type and *reil1-1 reil2-1* were determined as proxies of relative biomass accumulation at day 0, day 1, days 7 and 21 after cold shift using morphometric data (see Methods – parameterizing the objective function). Along with the morphometric data, the relative changes of metabolite pools and transcripts were profiled^30^ (see "Methods" section for details).

The experimental setup and the availability of transcriptomics data and data on relative metabolite levels allowed the application of TC-iReMet2^29,31^ to quantify the nominal and relative differences in metabolic fluxes of the wild type and the mutant (Supplementary Fig. S1). We refer to nominal changes as the sum of predicted flux differences, defined as the absolute value of difference between wild type and mutant flux, over all analyzed time points. The nominal changes may provide a skewed picture about the differences, particularly since the differences in fluxes between reactions in a given flux distribution differ in several orders of magnitude^20^. As a result, differences between fluxes that are anyhow small will be dominated by the differences between fluxes that take larger values. To remedy this issue, we also calculated the relative changes, defined as the sum of normalized flux differences over all analyzed time points, where the flux differences between wild type and mutant were normalized to their respective absolute maximum value over all time points. To apply TC-iReMet2 we used a bottom-up assembled model of *A. thaliana*, ArabidopsisCore^23^. This model consists of 549 reactions, of which 229 are transport reactions and artificial reactions representing growth (biomass) and non-growth-associated maintenance functions (NGAM^22^).

**Figure 2:**
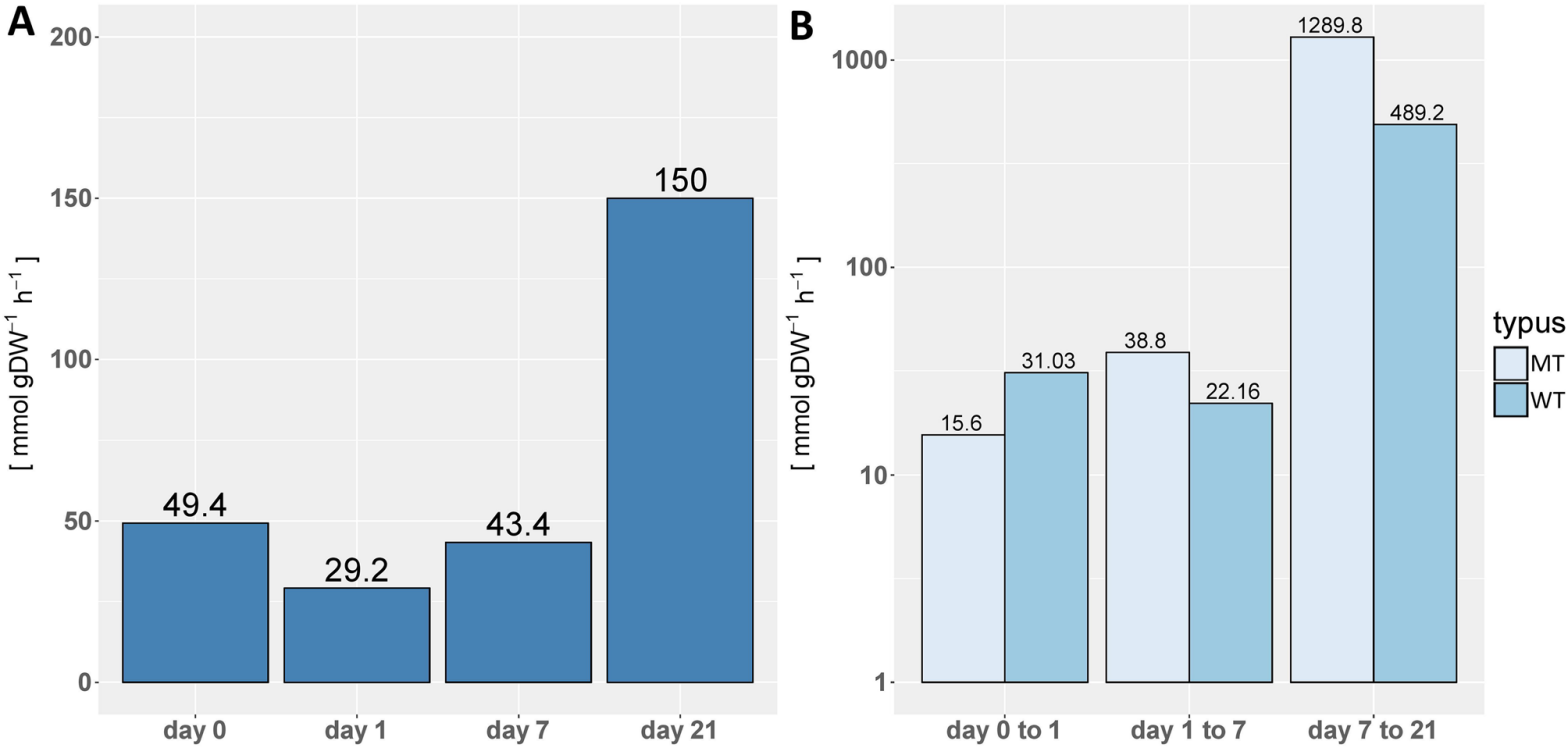
Changes in predicted sum of Fluxes. Shown are the optimal values of the Euclidean distance (displayed on y-axis) at each corresponding time point or time step (displayed on x-axis). Distances were visualized by plotting the Euclidean distance value above each bar. (**A**) Displayed are the sums of flux difference between wild type and mutant at each corresponding time point. (**B**) Displayed are the sums of flux differences between wild type fluxes and mutant fluxes between each two time consecutive points.

### Sum of predicted flux differences

The overall flux distance of wild type compared to mutant across all predicted reactions differed before cold shift, with the wild type having a higher overall flux (Fig. 2A). This prediction was consistent with the slight growth advantage of the wild type at the optimized growth temperature (Fig. 1). The difference of fluxes between consecutive time points remained approximately constant during the common hibernation phase, up to day 7. When the wild type resumed growth in the cold, the overall predicted flux differences increased approximately 3-fold. When considering the sum of flux changes per time step for wild type (Supplementary Fig. S2) and mutant (Supplementary Fig. S3), we find similar changes for the wild type and the mutant at the steps from 0 days to 1 day and 1 day to 7 days, with an increase in the change from day 7 to day 21 (Fig. 2B). However, we observe that the changes between day 7 and 21 are considerably larger in the wild type in comparison to the mutant, in line with the resumed growth of the former in the cold. In the following, we identify the reactions and pathways which contribute most to these observed differences.

### Analysis of differential reactions

We next considered the flux differences for each reaction in the metabolic model. Additionally, we investigated reactions displaying large changes in flux differences at early time points, as the most interesting to understand the changes in the metabolic network functionality in response to the cold shift.

#### K-means clustering of reaction behaviour

**Figure 3:**
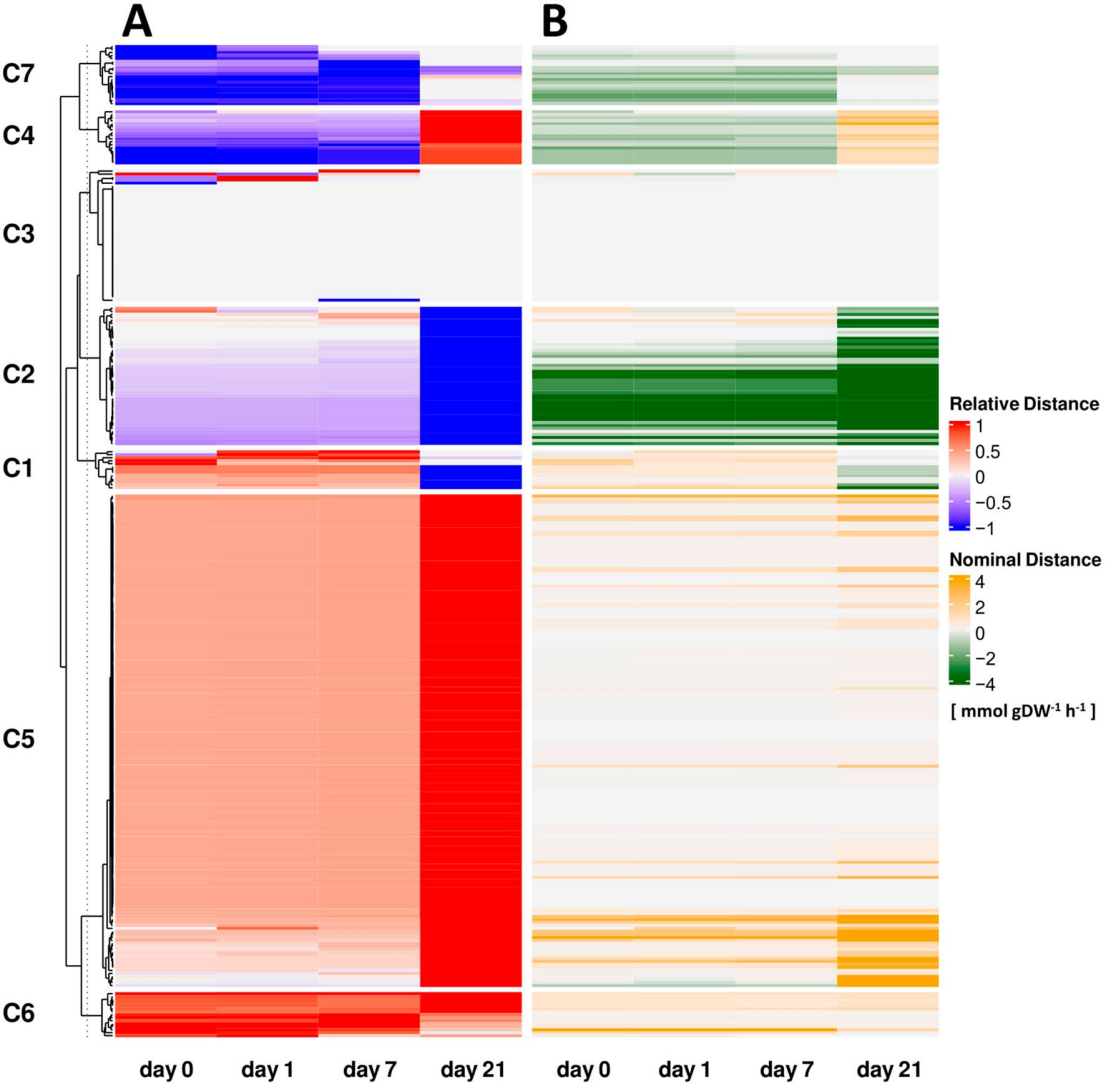
Overview of K-means clustering based on relative changes in reaction fluxes. K-means with Euclidean distance was used to identify seven clusters (C) of reactions (excluding transporters and artificial reactions). (**A**) Shows flux difference values normalized to the absolute maximum difference of each reaction for each time point. Corresponding nominal flux differences are shown in (**B**).

We focussed on differential behaviour of all reactions between mutant and wild type, excluding transport reactions and artificial reactions to avoid bias due to lack of gene association for these reactions. To this end, we applied K-means clustering to group reactions (Supplementary Table S2) with similar relative flux changes, where the number of clusters was determined by the silhouette index (Supplementary Fig. S4). As a result, we identified K = 7 clusters of reactions (Fig. 3), with a maximum silhouette index value of 0.78, based on the relative flux changes (Fig. 3A). For comparison, we also consider the K-mean clustering of the nominal flux changes (Fig. 3B). To provide an intuitive description of clusters as well as reaction behaviour over time, we introduce a three-character pattern consisting of Up (U), Down (D) and No changes (N) if the respective relative flux differences increased, decreased or stayed the same between two time points. Using this classification method we found 17 from the 27 possible patterns displayed by 320 reactions. A total of 111 reactions were classified by the most common pattern ’UUU’ making up roughly 35% of all observed patterns.

Overall, we mainly identified conserved flux differences in the first three time points with a shift in flux difference at day 21. This behaviour can be observed in the three biggest clusters. Cluster 5 consisted of 164 reactions, which exhibit an increase of relative flux changes (UUU). In contrast, cluster 2, consisting of 46 reactions, exhibited mainly decrease of relative flux changes (DDD). This inverse behaviour is best captured by the function of RuBisCO as it exhibits strong flux changes for its carboxylation function (cluster 5) and oxygenation function (cluster 2). Reactions in cluster 3 mainly exhibited no changes (NNN). Similarly, cluster 6 summarizes reactions that exhibit constant positive flux change over all time points. The remaining clusters 1, 4 and 7 group reactions that exhibit an inverse shift in behaviour at day 21.

If we consider the top 10 reactions (Supplementary Table S3) with respect to relative and nominal changes directly after cold shift, we find H-serine dehydrogenase (HSerDHNADP_h (UDU), HSerDHNAD_h(DUD)), isocitrate dehydrogenase (iCitDHNADP_m (DDD), iCitDHNAD_m(UUD)) as well as 6-phosphogluconic dehydrogenase (6PGDHNAD_h(DUD)), glutamate dehydrogenase (GluDH1NADP_m(DUD)) and glutamate synthetase (GluSNAD_h(UDD)) conserved among both measures. All these reactions are redox reactions. Additionally, 6-phosphogluconic dehydrogenase (6PGDHNADP_h(UDU)), glutamate dehydrogenase (GluDH2NAD_m(DUD)) and glutamate synthetase (GluSNAD_h(UDD)) can only be found in the top 10 reactions of nominal changes. Conversely, malate dehydrogenase (MalDH2NADP_c(UNN)), fructose-biphosphate aldolase (SBPA_h(UDD)) and sedoheptulose-biphosphatase (SBPase_h(UDD)) can only be found in the top 10 reactions of relative changes.

### Pathways enriched in reactions with highly altered fluxes across time points

**Figure 4:**
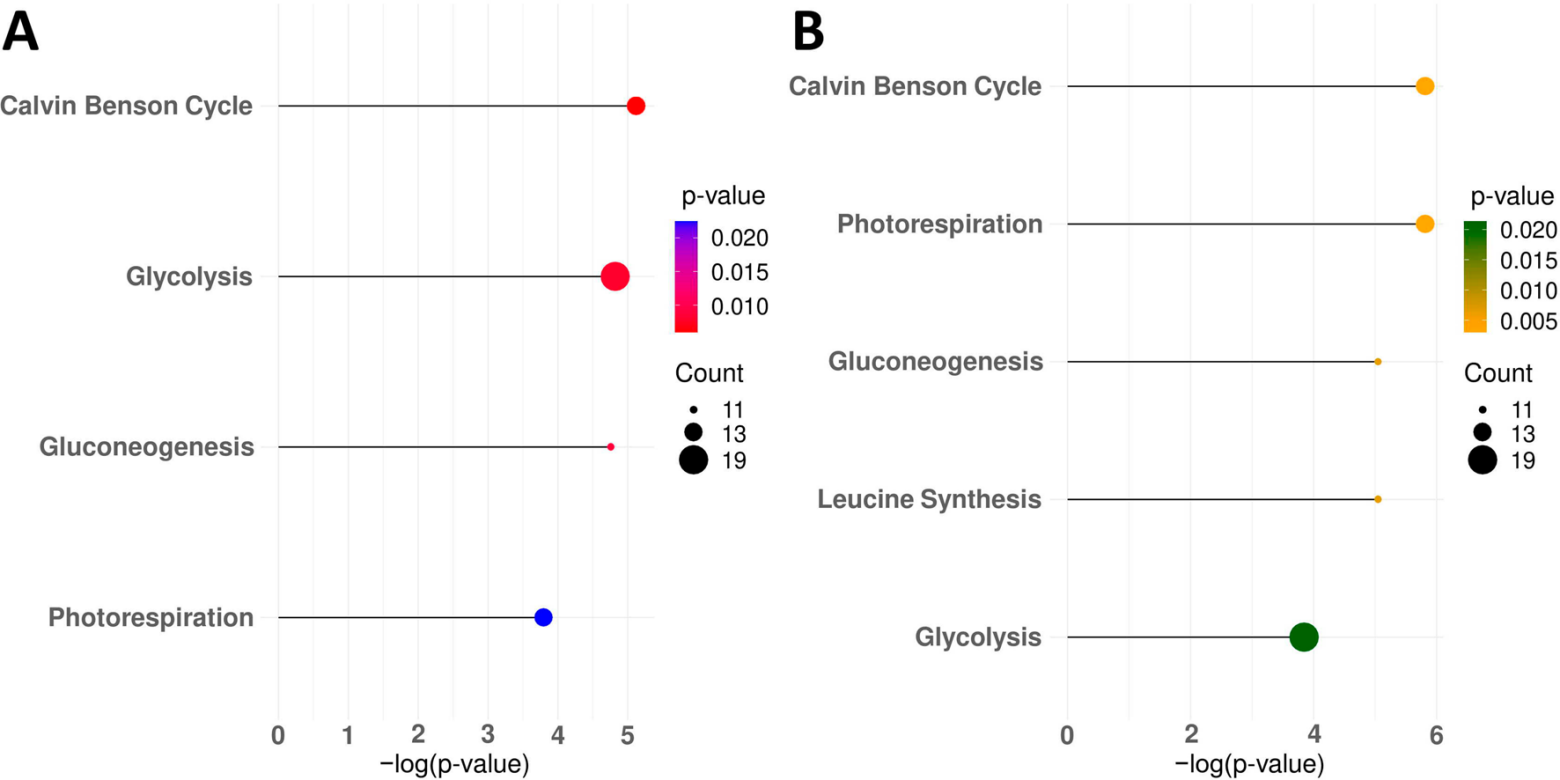
Pathways enriched in reactions with highly altered fluxes. Displayed are pathways significantly ($\textit{P} <= 0.05$) enriched in regulated reactions based on (**A**) relative and (**B**) nominal differences. They are descending ordered according to their respective *P*-value. Size of the dots corresponds to the count of reactions present in the pathway. Bar size represents the negative logarithm of the *P*-value (x-axis).

Metabolic reactions do not function in isolation, so analysis and interpretation of the prediction is best carried out in terms of pathways. To identify the pathways that are changed over time, we used the metabolic pathways as defined by the underlying *A. thaliana* model^23^(for definitions of pathway membership refer to Arnold et al. 2014^23^ (Supplementary Table S4). We inspected and considered as relevant those pathways that were enriched with reactions displaying large predicted flux differences between wild type and mutant (Fig. 4). A reaction was defined to exhibit large changes, if its absolute sum of flux changes across all time points was above the median of considered reactions present in the model (excluding transport and artificial reactions, as specified above). To identify pathways enriched with such reactions we used the Fishers exact test with significance threshold *P* considering multiple hypotheses correction following the Benjamini–Hochberg procedure (Supplementary Table S5). Considering nominal changes, we found five pathways to be enriched for reactions with large changes. These pathways, ordered by decreasing *P*-value, with $p<$ 0.01, include: the Calvin–Benson-Cycle (CBC), photorespiration, gluconeogenesis, leucine synthesis, and in addition with $< 0.05$, glycolysis. Considering relative instead of nominal changes, we found pathways with $p < 0.01$ to include the Calvin–Benson cycle, glycolysis, gluconeogenesis, and in addition with $p < 0.05$, photorespiration.

### Flux sampling analysis with quadratic constraints

We examined how specific these findings are by sampling the solution space given in optimal solution for each considered time point. As a sufficiently large enough sample size gives information about range of fluxes as well as their probability, it gives the means to explore for alternative solutions and so for the uniqueness of the solution. In this case for each considered time point the proposed approach (see Methods - Flux sampling for TC-iReMet2) did not find a solution after 1000 trials. Therefore, this analysis indicates that the findings are specific, in the sense that alternative optima are unlikely, and significant as there are no other possible flux distributions in optimal solution.

## Discussion

Here, we proposed a computational approach, termed TC-iReMet2, and showed that it provides the means for time-resolved predictions of fluxes while keeping the simplicity of the constraint-based modelling framework and allowing for the integration of relative metabolomic, transcriptomic, and morphometric data. The findings of this study indicate that TC-iReMet2, a differential flux profiling method, can be used to identify differential fluxes between wild type and mutants over time. It is important to note that TC-iReMet2 uses the ratio of transcripts as a proxy for the ratio of enzyme abundance (following GPR rules). This is a strong assumption, knowing that post-translational modifications and translational efficiency have a large effect on both the abundances and ratios of proteins. However, since transcript ratios are used as one component of the constraints, such an approach provides a better coverage of metabolic networks than proteomics data^14^. With the advances in the proteomics profiling, TC-iReMet2 has the potential to provide further applications closer to the assumptions of the approach.

Moreover, the enzyme kinetic assumed in TC-iReMet2 does not consider saturation effects no presence of regulators (e.g. activators or inhibitors) of enzyme activity. Inclusion of a saturation effect, like in Michaelis-Menten kinetic, would not allow casting the problem with only linear constraints, rendering application to large-scale networks computationally challenging. Similar problem arises when considering the inclusion of regulation, which is additionally problematic due to the lack of information on how the effect of the regulator is captured in the enzyme kinetic form used, particularly for plants^32,33^. One possible approach to overcome this issues is to use a power-law formalism^34^, which would allow the constraints to remain linear, at the cost of making assumptions about which regulators affect a reaction rate and with what strength. For this reasons we have decided that TC-iReMet2 is formulated based on mass-action-like kinetic, while allowing for discrepancy to model possible effects due to the mentioned saturation and regulation.

Applying TC-iReMet2 to the comparison of growth deficient *reil1-1 reil2-1* double mutant to *A. thaliana* Col-0 wild type before and after cold shift strongly supported for the previously suggested theory stating that REIL mediated ribosome biogenesis deficiency feeds back into metabolism. Overall, we find that flux differences are more similar during hibernation phase, with strong flux redistributions occurring at day 21. This is evident from the data from the morphometric analysis (Fig. 1, Supplementary Table S6). Mutant and wild type plants grow similar but start to differ strongly between days 7 and 21 after temperature shift.

Even more important, TC-iReMet2 enables a way to compare wild type and mutant differential fluxes prior to cold shift and in the early hibernation phase. Thus, it allows for the analysis of the mutant system relative to wild type without being obscured by the effects of differential growth occurring between days 7 and 21 of the current experiments. When considering differential fluxes during the hibernation phase, we find that REIL mediated ribosome biogenesis deficiency might feed back into metabolism by altering the RuBisCO carboxylase to oxygenase ratio (Supplementary Table S7). Additionally, mutant associated deficiencies of the CBC and glycolysis fluxes combined with mutant-specific increase of all fluxes in the photorespiratory pathway support this hypothesis (Fig. 4). Overall the strongest mutant flux deficiencies appear to be in the RuBisCO (carboxylation), FNR, malate dehydrogenase and alanine transaminase reactions (Supplementary Table S5).

Predicted relative flux changes directly after cold shift appear to be small. However, one day after cold shift, the fluxes of reactions distributed across various pathways of central metabolism, including carbohydrate, organic acid and amino acid metabolism differ between mutant and wild type. What is common to these reactions is that they all require NAD(P) as a cofactor. These changes may indicate either an altered redox state of these cofactors or more likely differential use of NAD and NADP after cold shift in the mutant. For example, when considering the predicted inverse flux changes of the mitochondrial iCit dehydrogenase isozyme reactions, iCitDHNAD_m and iCitDHNADP_m, we can deduce in agreement with our metabolic model that the mutant switches to preferential use of NAD rather than NADP for this reaction. Inversely, a preferential use of NADP is predicted for 6-phosphogluconic dehydrogenase reactions, 6PGDHNADP_h and 6PGDHNAD_h, and for the H-serine dehydrogenase isozyme-reactions, HserDHNADP_h and HserDHNAD_h. Taken together with the additional indicated flux changes of glutamate synthetase (GluSNAD_h), and of the mitochondrial malate dehydrogenase (MalDHNAD_m), or glutamate dehydrogenases, GluDH1NADP_m and GluDH2NAD_m, we hypothesize that the *reil1-1 reil2-1* double mutant defect is associated with a NAD/ NADP cofactor deregulation.

Generation of this hypothesis would not have been possible by stand-alone analysis of the transcriptome data alone. When we compare the results of TC-iReMet2 with a differential analysis of the transcriptomics data reaction per reaction following GPR rules, only the increase of isocitrate dehydrogenase (iCitDHNAD_m) flux in the mutant can be found overlapping with TC-iReMet2’s predictions (Supplementary Table S8). This indicates that TC-iReMet2’s integration of metabolomics and transcriptomics data provides added value compared to the sole analysis of either, thus, allowing new and additional support for hypothesis generation. Verification of these findings and hypothesis testing can be performed by subsequent studies and detailed quantification of NAD and NADP levels and their redox states under same and extended experimental set-ups. Altogether, the predictions from TC-iReMet2 suggest that altered use of NAD and NADP or of their redox state is an important mechanism by which REIL mediated ribosome biogenesis deficiency feedbacks into metabolism early after cold shift.

The current formulation of TC-iReMet2 has the potential to be further optimized, since the weighting parameters of the objective function are chosen based on the assumption that the consecutive increase between time points equals consecutive decrease of weighting. Validation of predictions gave robustness to this assumption. Yet, the usage of different weights could be considered based on other insights from independent physiological measurements. In addition, rather than using relative transcriptomics data, relative proteomic data^9^ or enzyme activity measurements^35^ could be integrated to provide more reliable predictions that are less influenced or obscured by post-transcriptional levels of regulation than transcriptome data. Therefore, TC-iReMet2 improves existing constraint-based approaches for differential flux prediction by accounting for possible temporal physiological change while also allowing for the integration of morphometric data.

## Methods

### Flux sampling for TC-iReMet2

Uniform flux sampling provides an unbiased characterization of the solution space. When enough flux distributions are sampled, they can be used to analyze their probability distributions or the range of specific fluxes. In this setup, flux sampling was performed using a random walk algorithm (Hit and Run).

A linear program with a single quadratic constraint is defined to find possible alternative solutions. For this, the solution space has to be defined. In addition to the defining constraints given by TC-iReMet2, a single quadratic constraint, due to quadratic objective function TC-iReMet2 is based on, has to be introduced. It fixes the value of the objective function to be the same as in optimal solution, forcing the optimization to find alternative optima. Here, *z* denotes the value of TC-iReMet2’s objective function and $z*$ the value found in optimal solution at the specific time point. Fluxes at the current time point are denoted by $v_{t+1}$, whereas $v_{t}$ denotes the flux distribution of the prior time point. We also allow for a small deviation, denoted by $\zeta$, of the objective function at the optimum to counteract numerical problems. This way the solution space, containing all possible solutions at the time point specific optimum, is defined. To sample this space, steps are done as follows:

$$\begin{aligned} \begin{array}{ll@{}ll} {\displaystyle \max _{\lambda , v_{t+1}, z} \lambda } &{} &{} \\ \\ {s.t.}&{} &{} &{} \\ &{} &{} &{} \\ \\ {Sv_{t+1}^{A}=Sv_{t+1}^{B} = 0 ,} \\ \\ {v_{min}^{A} \le v^{A}_{t+1} \le v_{max}^{A},} \\ \\ {v_{min}^{B} \le v^{B}_{t+1} \le v_{max}^{B},} \\ \\ { (\varkappa _{t+1}-\delta )\;opt^A \le v_{Biomass}^{A} \le (\varkappa _{t+1}+\delta )\;opt^A ,} \\ \\ {v_{biomass}^B = opt^B , }\\ \\ {\forall i \in \mathfrak {I}: v_i^{B}q_{i}\prod _{j\in F(i)}(r_j^{min})^{|S_{ji}|}-\varepsilon _i \le v_i^{A} \le v_i^{B}q_{i}\prod _{j\in F(i)}(r_j^{max})^{|S_{ji}|}+\varepsilon _i,} \\ \\ {\forall j \in \{ \ell | \chi (r_\ell )=1\}:r^{min}_j=\hat{r}_j^{min}, r^{max}_j=\hat{r}_j^{max} ,} \\ \\ { \forall j \in \{ \ell | \chi (r_\ell )=0\}:r^{min}_\ell =min_{m:\; m \in \{ \ell | \chi (r_\ell )=1\}}\;{{\hat{r}}_m^{min}}, \;\; r^{max}_\ell =max_{m:\; m \in \{ \ell | \chi (r_\ell )=0\}}\;{{\hat{r}}_m^{max}} ,} \\ \\ {\forall i \in \{ \eta | H (q_\eta )=1\}:q^{min}_i=\hat{q}_i^{min}, q^{max}_i=\hat{q}_i^{max} ,} \\ \\ {\forall i \in \{ \eta | H (q_\eta )=0\}:q^{min}_\eta =min_{m:\; m \in \{ \eta | H (q_\eta )=1\}}\;{{\hat{q}}_m^{min}}, \;\; q^{max}_\eta =max_{m:\; m \in \{ \eta | H (q_\eta )=0\}}\;{{\hat{q}}_m^{max}} ,} \\ \\ {0 \le \lambda \le \infty ,} \\ \\ { (1-\zeta )z*\le z \le (1+\zeta )z* ,} \\ \\ { v_{t+1} = v_{0} + \lambda \;v_{direction} ,} \\ \\ { z = \alpha ||(v^{A}_{t+1}-v^{B}_{t+1})||_2^{2}+\beta ||(v^{A}_{t+1}-v^{A}_{t})||_2^{2}+\gamma ||(v^{B}_{t+1}-v^{B}_{t})||_2^{2}} \end{array} \end{aligned}$$

1. Select an initial point $v_0$ in solution space (here, we used $v_{t+1}$ in optimal solution, derived from the main optimization problem of TC-iReMet2 as $v_0$, since it must lie in the solution space).

2. Select a random direction $v_{direction}$ pointing in solution space.

3. Find the extreme point in solution space described by $v_{t+1} = v_0 + \lambda \; v_{direction}$ by solving a linear program with a quadratic constraint (due to the quadratic problem the main objective is based on): If there is no solution to the optimization problem given above, $v_{direction}$ does not point into solution space. As a consequence, steps 2. and 3. are repeated until a solution is found.

4. If there is a solution for the optimization program at step 3, a new point at the edge of the solution space can be determined to form a line segment with the initial point.

5. Randomly choose a point on this line segment to create a sample, which is in turn updated as a new initial point $v_0$.

6. Repeat steps 1. to 5. until the defined number of samples is collected.

### Numerical stability of TC-iReMet2

The multiplication of relative metabolite levels, substitution of unmeasured metabolite ratios with their respective minimum and maximum ratio value together with approximated enzyme ratios can lead to immense ratio constraints, which in turn could lead to numerical instabilities. Determining a maximum considered ratio constraint is therefore crucial to ensure numerical stability. To this end, we calculated the flux distributions allowing for a maximum ratio constraint ranging from 10$^1$ to 10$^{21}$ (includes the maximum possible ratio constraint in this setup) 10 times. Since each of those repeated measurements resulted in the same flux distribution, we used Pearson correlation to measure similarity between each flux distribution to its prior and successive one. Overall, correlations between flux redistributions were very high being above 0.9. The highest correlated region, while having a feasible solution at each considered time point, was detected when allowing for a maximum ratio constraint of 10$^8$. This leads to 252, 252, 253 and 189 ratio constraints made at each considered time point. Hence, no ratio constraint exceeding 10$^8$ was considered in this setup to ensure numerical stability.

### K-means clustering

We used R statistical programming languages implementation of the K-means algorithm with seven assumed clusters ($K=7$) and Euclidean distance as distance measure. The reason for selecting $K=7$ is the following: We assumed there to be one cluster of no differences fluxes, one cluster displaying stronger wild type flux with a rise at 21 days and inversely the same for mutant. Two clusters of inverse behaviour where wild type or mutant flux is stronger at the first time points with a shift in sign at 21 days. Lastly, we assumed two clusters of consistent flux difference favouring wild type or mutant conserved over all time points. This line of reasoning was supported by the silhouette index analysis, which specify the number of clusters K = 7 to maximize the value of the index.

### Fishers exact test for enrichment analysis

We used a right-tailed Fisher’s exact test to determine the enrichment in regulated reactions of a pathway. To this end, we defined a reaction as regulated if its sum of relative or nominal differences across time points was above its corresponding median of all considered reactions, else we considered the reaction to be unregulated. Therefore, we tested the association between regulated reactions and pathways for both relative and nominal differences. This test was conducted with a significance level of 0.05 through Matlab’s ’fishertest’ function. The resulting *P*-values were corrected for multiple hypotheses testing following the Benjamini–Hochberg procedure.

### Parameterizing the objective function of TC-iReMet2 and estimating fractions of biomass yield

The analyzed time points differed in scale and therefore constituted a good case to test the assumption that a subsequent flux distribution is dependent on the previous one, therefore allowing for different magnitudes of physiological change. To model the dependency of flux distributions between time points, we assumed a steady decrease in the dependence as the interval between the points increases. More specifically, the following weights were used at each time point depicted by Table 1. Weighting of flux distribution dependency of scenario *A* and *B* to a prior one is denoted by $\beta$ and $\gamma$ respectively. Difference of flux distributions between scenario *A* and *B* at analyzed time point was weighted by $\alpha$.

**Table 1:** Weight values of TC-iReMet2’s objective function and biomass fraction values at each analyzed time point.

Time point	$\alpha$	$\beta$	$\gamma$	$\varkappa$
day 0	1	0	0	0.77
day 1	0.34	0.33	0.33	0.77
day 7	0.7	0.15	0.15	0.76
day 21	0.9	0.05	0.05	0.51

To model the fraction of biomass yield in scenario *A* to the biomass yield in scenario *B* at each specific time point $\varkappa _{t+1}$, we used four biomass proxy parameters (Supplementary Table S1), two diameter measurements of the *A. thaliana* rosette, diameter 1 and 2, the apparent planar leaf area, and leaf perimeter (i.e. the circumference). The morphometric parameters apparent planar leaf area and perimeter underestimate biomass accumulation, since rosette leaves could slightly overlap. We contrasted these estimates by using the sum of diameter 1 and 2 as proxies of biomass accumulation. The diameter may slightly overestimate biomass because only the longest leaves are considered. Accordingly, we integrated the four biomass proxy parameters by giving equal weight to each one of them (Supplementary Table S1). In detail, the morphometric parameter measurements were averages separately for the wild type and the mutant. Ratios of mutant and wild type were calculated per time point based on the averages of biomass proxies. Finally, the resulting ratios across the four biomass proxies were averaged to obtain time point specific biomass fractions $\varkappa _{t+1}$. We allowed to a deviation $\delta$ of +/- 0.05 from the biomass fraction $\varkappa _{t+1}$ as these calculations are estimates. In absence of biomass estimates, e.g. at day 1, we assumed a steady decrease of biomass fraction $\varkappa _{t+1}$ between day 0 and day 7. Therefore, the ratio at day 1 is a seventh closer to the biomass fraction $\varkappa _{t+1}$ of day 7 compared to day 0, resulting in a fraction of 0.77. All used biomass fractions $\varkappa _{t+1}$ are depicted in Table 1.

### Transcriptomics and metabolomics data used

The transcriptomics data are already published and are uploaded to the Gene Expression Omnibus (https://www.ncbi.nlm.nih.gov/geo/) and are available through accession number GSE101111. The metabolomics data are obtained from the Supplemental Table S1 of Beine-Golovchuk et al., 2018^30^. The morphometric data are obtained from Schmidt et al., 2013^2^. All data are included in the provided GitHub repository (https://github.com/tciremet2/TC-iReMet2) as well as in the Supplementary Tables to ensure easy access and reuse of the provided implementation.

### Implementation and tools

For implementation of TC-iReMet2 we used “MATLAB 2017b, The MathWorks”^36^ in conjunction with the Tomlab optimization environment^37^. Statistical analysis and creation of figures was done with R^38^ programming language and R’s ggplot2 library^39^ and “MATLAB 2017b, The MathWorks”^36^. The implementation is available at https://github.com/tciremet2/TC-iReMet2.

## Supplementary Information

Supplementary Information 1.

Supplementary Information 2.

## Data Availability

The transcriptome data are available from the Gene Expression Omnibus (https://www.ncbi.nlm.nih.gov/geo/) through accession number GSE101111. The metabolome data are previously published among the supplemental data of Beine-Golovchuk and co-authors^2,3^.
