# Supplementary material for: Integration of relative metabolomics and transcriptomics time-course data in a metabolic model pinpoints effects of ribosome biogenesis defects on Arabidopsis thaliana metabolism
Source: Sci Rep. 2021 Feb 26;11:4787. doi: 10.1038/s41598-021-84114-y (PMC7910480; doi:10.1038/s41598-021-84114-y)
Supplement: Supplementary file 2 — Supplementary Information 2. [file 41598_2021_84114_MOESM2_ESM.pdf]

Integration of relative metabolomics and transcriptomics time-course data in a metabolic model pinpoints effects of ribosome biogenesis defects on *Arabidopsis thaliana* metabolism: Supplementary note

Christopher Pries, Zahra Razaghi-Moghadam, Joachim Kopka, Zoran Nikoloski

In this note, we provide further information about the flux differences between wild type and mutant for each reaction (Supplementary Figure S1) and the differences in fluxes between times points for wild type (Supplementary Figure S2) and mutant (Supplementary Figure S3) fluxes for each reaction. Additionally, we supply silhouette plots (Supplementary Figure S4) to support the selected number (k) of clusters for the k-means clustering of reaction behaviour.

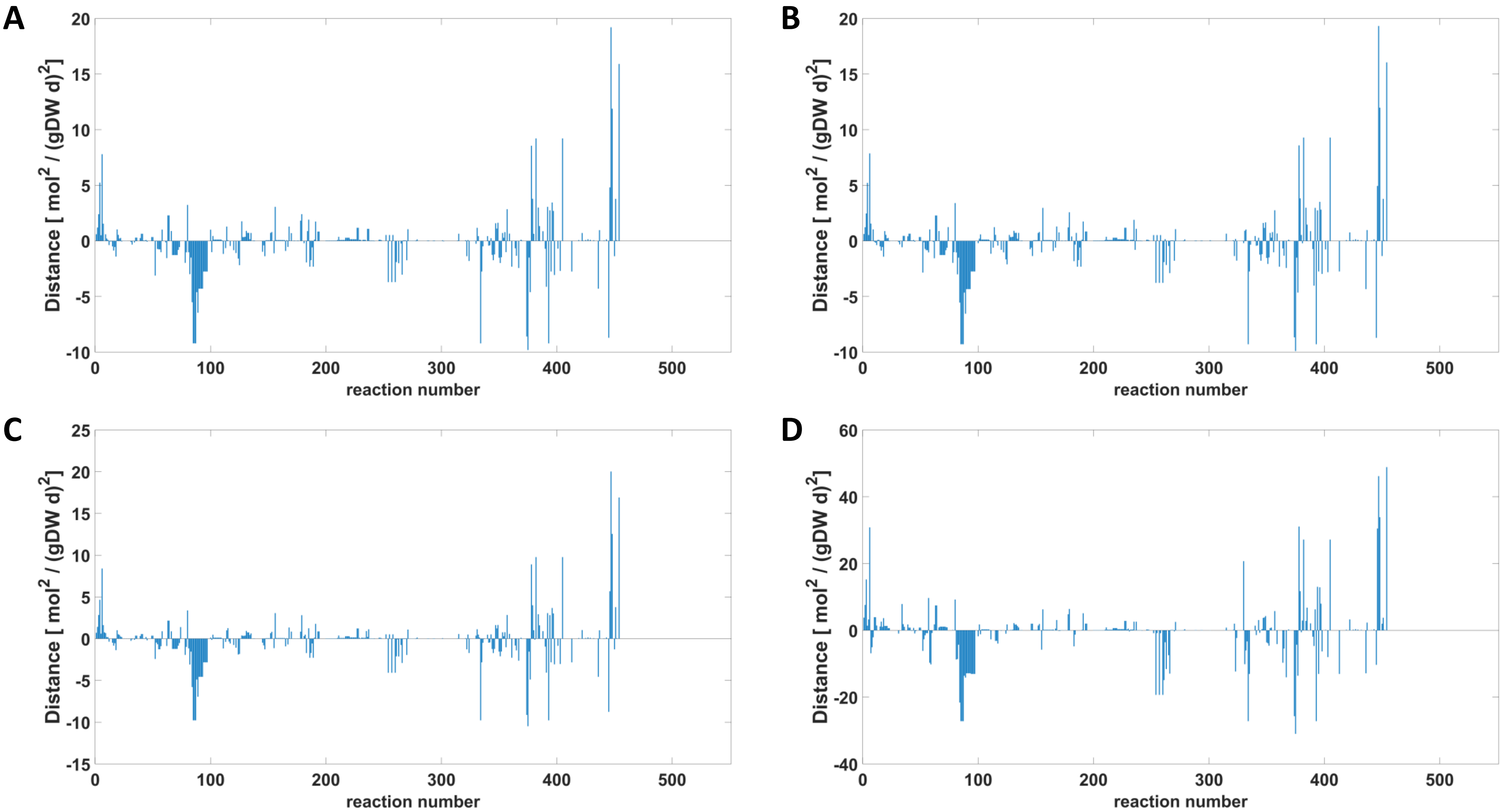

Supplementary Figure S1. Changes in flux differences per reaction. Shown is the flux differences between predicted wild-type and mutant fluxes (y-axis) of each reaction (x-axis) based on the optimal values of the Euclidian distance at time points day0 (A), day1 (B), day7 (C) and day21 (D).

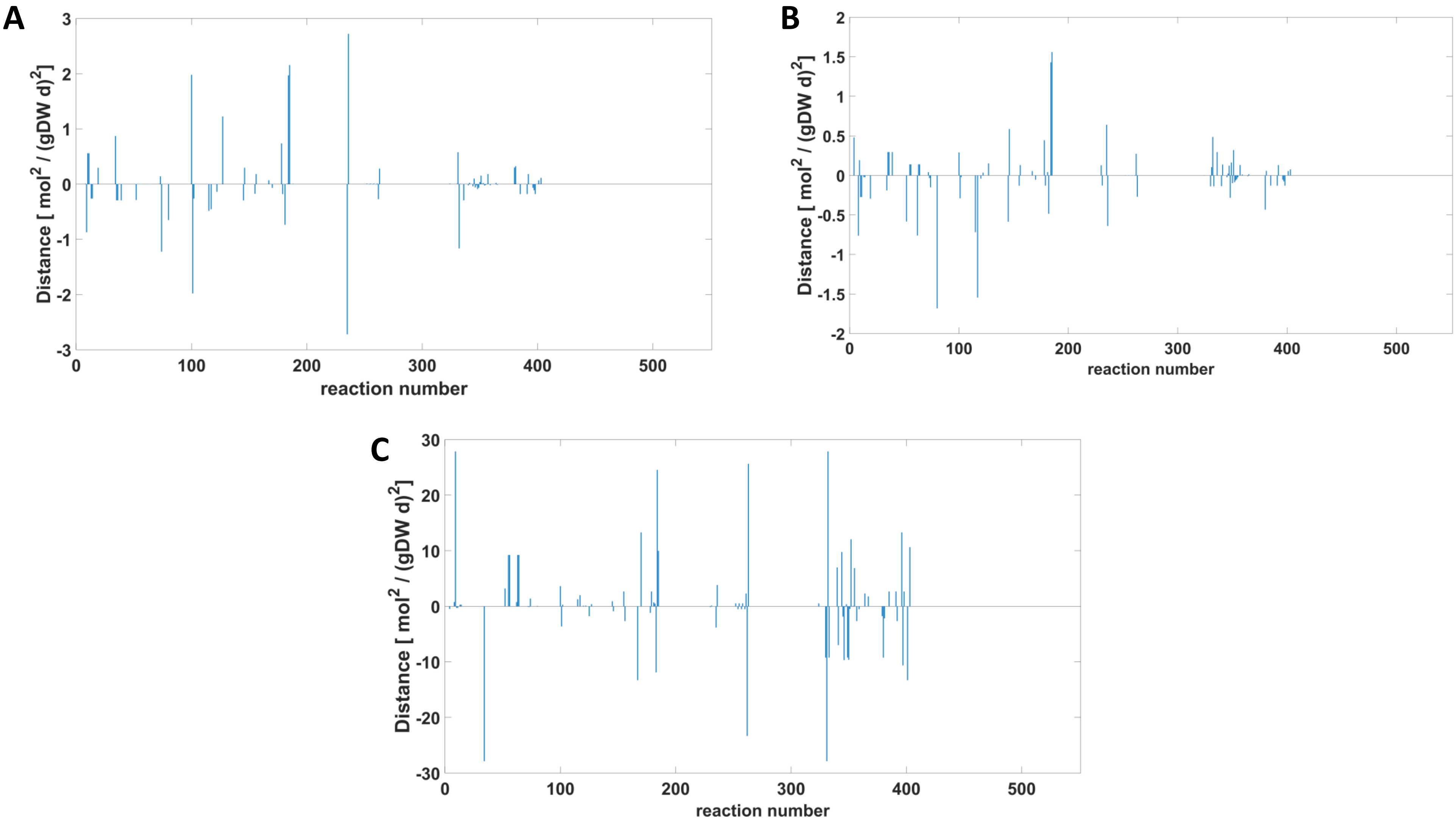

Supplementary Figure S2. Changes in wild-type flux differences per time step. Displayed are the flux differences (y-axis) between wild-type fluxes of each reaction (x-axis) between each two time consecutive points with day0 to day1 (A), day1 to day7 (B) and day7 to day 21 (C).

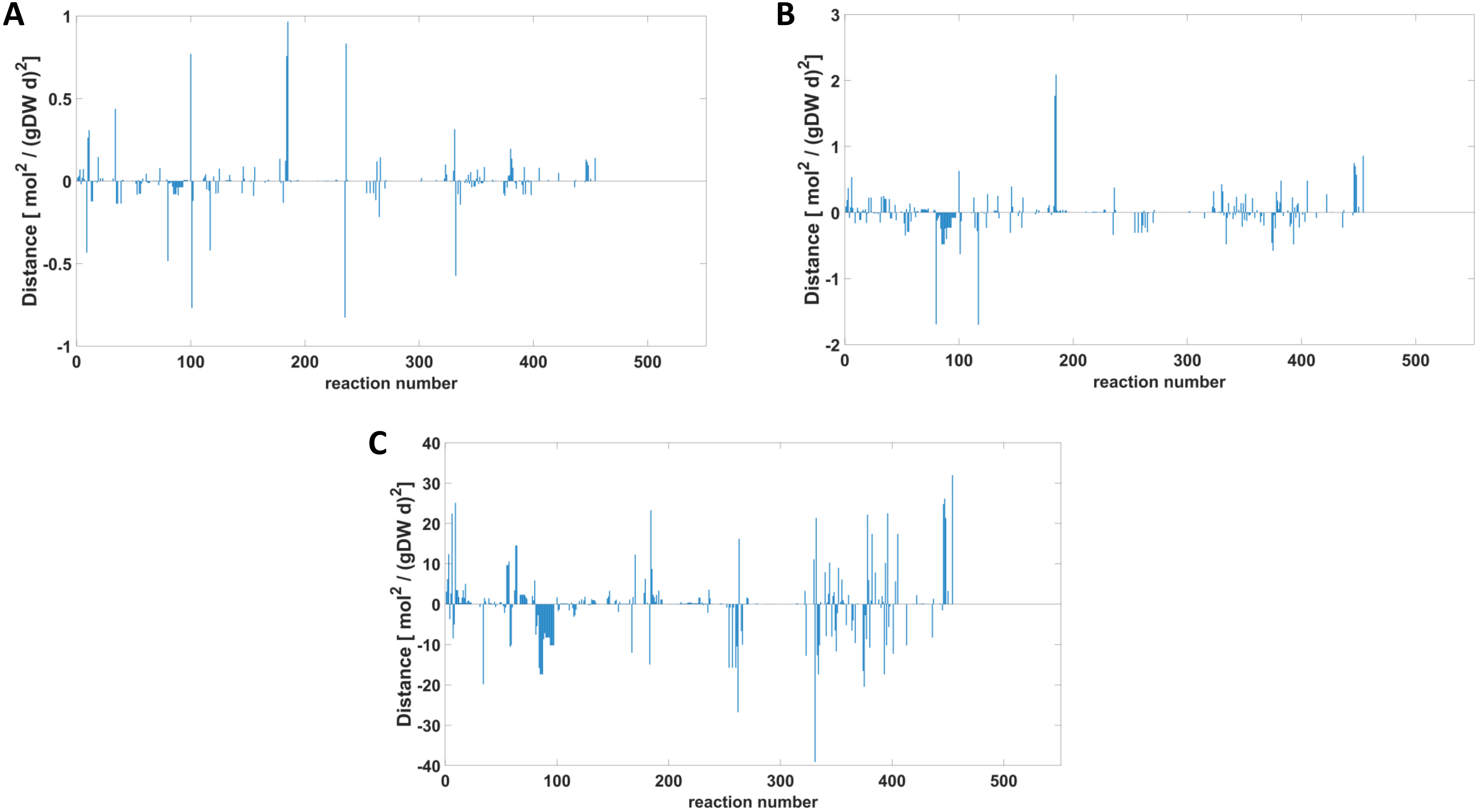

Supplementary Figure S3. Changes in mutant flux differences per time step. Displayed are the flux differences (y-axis) between mutant fluxes of each reaction (x-axis) between each two time consecutive points with day0 to day1 (A), day1 to day7 (B) and day7 to day 21 (C).

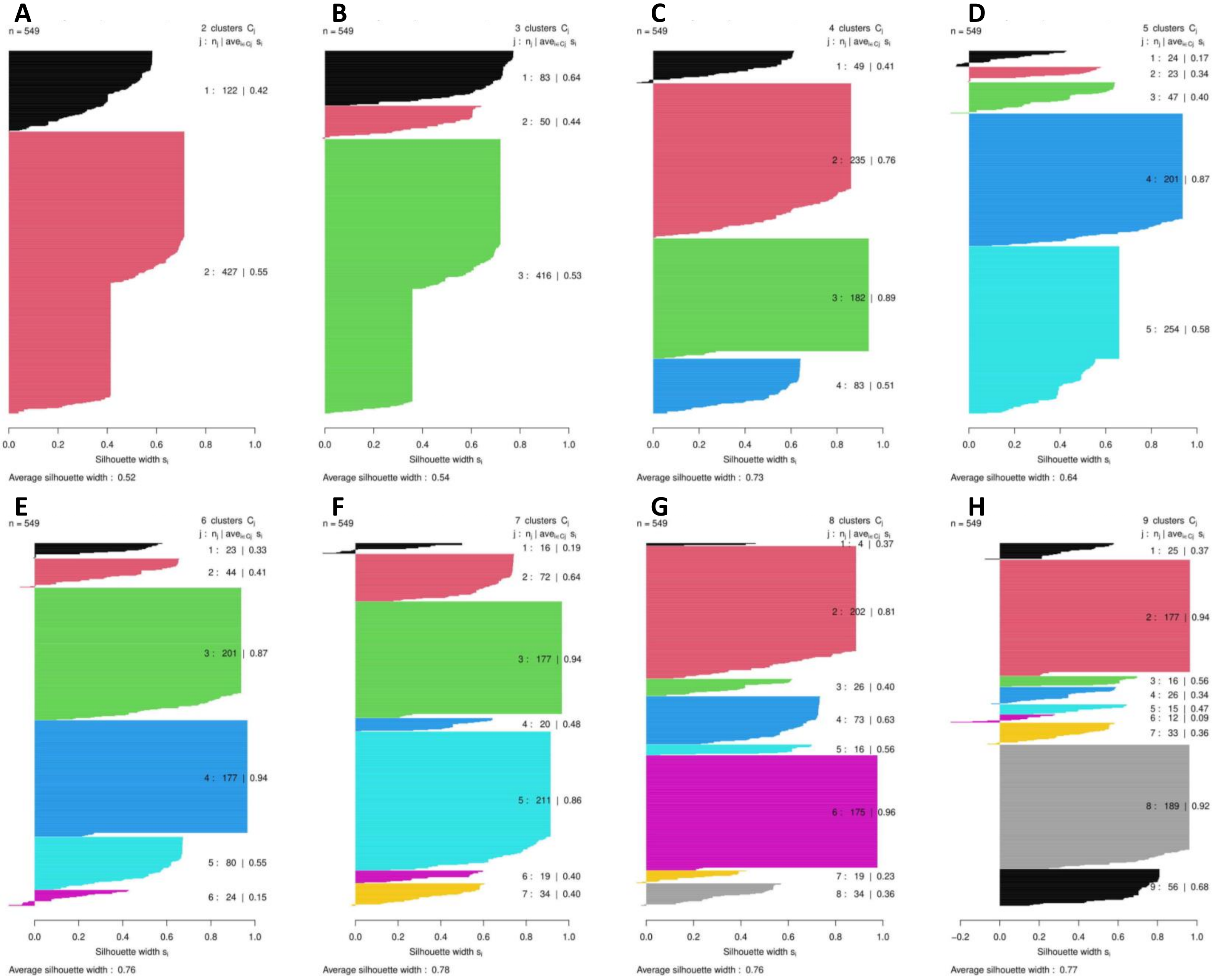

Supplementary Figure S4. Silhouette index analysis. Displayed are the silhouette plots based on k-means clustering with k=2(A), k=3(B), k=4(C), k=5(D), k=6(E), k=7(F), k=8(G) and k=9(H). The bar length corresponds to the silhouette width of each respective reaction given by the cluster (x-axis). The average silhouette width is displayed on the bottom of each plot. The colors indicate the cluster membership of each displayed reaction. On the right of each cluster the cluster number, count of reactions and the average width of the cluster are displayed (clusternumber: count of reaction | average width). On the top left the total count of reactions (n) is displayed.
